# A Pandemic in Times of Global Tourism: Superspreading and Exportation of COVID-19 Cases from a Ski Area in Austria

**DOI:** 10.1128/JCM.00588-20

**Published:** 2020-05-26

**Authors:** Carlos L. Correa-Martínez, Stefanie Kampmeier, Philipp Kümpers, Vera Schwierzeck, Marc Hennies, Wali Hafezi, Joachim Kühn, Hermann Pavenstädt, Stephan Ludwig, Alexander Mellmann

**Affiliations:** a Institute of Hygiene, University Hospital Münster, Münster, Germany; b Department of Medicine D, Division of General Internal Medicine, Nephrology, and Rheumatology, University Hospital Münster, Münster, Germany; c Institute of Virology, University Hospital Münster, Münster, Germany; Boston Children's Hospital

**Keywords:** Austria, COVID-19, SARS-CoV-2, exported cases, pandemic, superspreading, tourism

## LETTER

On 7 January 2020, the World Health Organization (WHO) announced a novel coronavirus to be the cause of pneumonia cases whose cause was unclear in China. The infection came to be known as a coronavirus disease (COVID-19) caused by severe acute respiratory syndrome coronavirus 2 (SARS-CoV-2). After the disease spread to 114 countries, a COVID-19 pandemic was declared by WHO on 11 March (https://www.who.int/emergencies/diseases/novel-coronavirus-2019).

**FIG 1:**
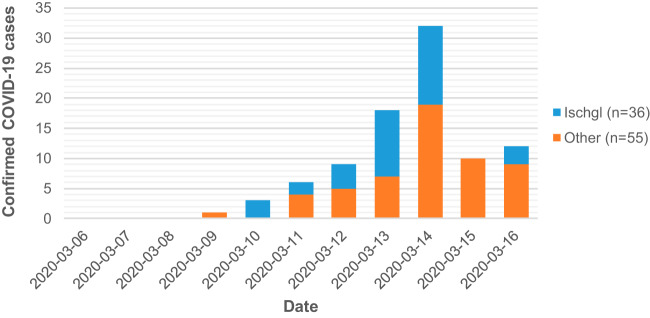
Geographical origin of SARS-CoV-2 diagnosed at UKM between 6 March and 16 March 2020.

Between 9 and 16 March, increasing numbers of COVID-19 cases were detected at University Hospital Münster (UKM), a tertiary care center in northwestern Germany. Of 90 patients, 36 had recently visited Ischgl (39.6%) (Fig. 1), a popular ski town in the Austrian Alps. With 22,626 beds for visitors, 492,798 tourists arrived in the 2018/2019 season, including guests from over 20 different countries. American tourists represented the most relevant group of non-European guests, with 6,886 overnight stays (https://www.tirol.gv.at/statistik-budget/statistik/publikationen/).

**FIG 2:**
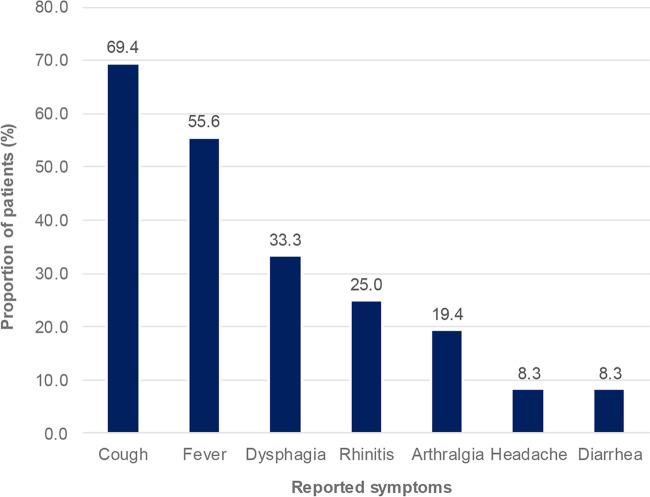
Symptoms displayed by SARS-CoV-2-positive patients consulting at UKM upon their return from Ischgl, Austria.

Several of our patients had visited après-ski bars in Ischgl, and some recalled having contact with subjects with subsequently confirmed cases. Patients were predominantly male (61.1%) and aged 20 to 71 years (mean, 43.3). All displayed symptoms, including cough (69.4%), fever (55.6%), and dysphagia (33.3%) (Fig. 2).

Quantitative real-time PCR analysis of nasopharyngeal swabs was performed (1), yielding positive SARS-CoV-2 results in 36 Ischgl cases. Another nine Ischgl tourists had equivocal test results, implying a potentially higher proportion of Ischgl-related COVID-19 cases. Thus, we included Ischgl as a risk region in our internal COVID-19 case definition, which was officially recommended by the German authorities 2 days later.

Iceland declared Ischgl a risk region as early as 5 March after travelers returning from a ski trip tested positive upon arrival (https://www.landlaeknir.is/um-embaettid/frettir/frett/item39457/Skidasvadid-Ischgl-i-Austurriki-i-hop-skilgreindra-ahattusvada). Norway reported 161 cases imported from Austria, representing 57.1% of all imported cases (https://www.fhi.no/en/id/infectious-diseases/coronavirus/daily-reports/daily-reports-COVID19/). In Denmark, this proportion reached 50% (https://files.ssi.dk/COVID19-overvaagningsrapport-18032020). On 7 March, an employee of a popular après-ski bar in Ischgl tested positive for COVID-19. However, local authorities estimated the transmission to visitors to be very unlikely (https://www.tirol.gv.at/meldungen/meldung/artikel/erhebungen-zu-am-coronavirus-erkrankten-norweger-im-bezirk-landeck-weiter-im-gange/). As cases continued to increase in number, a quarantine was declared in Ischgl on 13 March (https://www.bundeskanzleramt.gv.at/bundeskanzleramt/nachrichten-der-bundesregierung/2020.html) and the ski season was finally terminated on 14 March (https://www.ischgl.com/news-de). The local public prosecutor’s office then initiated an investigation of allegations of unreported COVID-19 cases diagnosed in late February in Ischgl (https://www.apa.at/Site/News.de.html?id=6353870430).

Our findings support European data indicating the exportation of COVID-19 cases from a cluster in Ischgl. The après-ski bar where the first patient diagnosed with COVID-19 in Ischgl worked as a barkeeper was reportedly the source of many of the cases later detected in Iceland, Norway, and Denmark (https://www.spiegel.de/politik/ausland/coronavirus-ausbruch-in-ischgl-die-brutstaette-a-8f56e5a2-635f-473a-96e9-300b6cbf4180, https://www.bbc.com/news/world-europe-52007104). Several of our patients also reported having visited the bar. This supports the theory of a superspreading event taking place in Ischgl, a phenomenon already observed in past SARS and Middle East respiratory syndrome (MERS) outbreaks (2–4). Considering the delayed epidemiological response, we believe that Ischgl-related cases continue to circulate undetected within and outside Europe. European travelers were banned from entering the United States on 14 March (https://www.whitehouse.gov/presidential-actions/?issue_filter=healthcare), 9 days after Iceland issued its epidemiological warning. An unknown number of infected travelers from Ischgl could thus have entered American territory in this time span, as observed in Germany and other European countries.
